# Activation of Parallel Fiber Feedback by Spatially Diffuse Stimuli Reduces Signal and Noise Correlations via Independent Mechanisms in a Cerebellum-Like Structure

**DOI:** 10.1371/journal.pcbi.1004034

**Published:** 2015-01-08

**Authors:** Benjamin Simmonds, Maurice J. Chacron

**Affiliations:** Department of Physiology, McGill University, Montreal, Quebec, Canada; Queen's University, Canada

## Abstract

Correlations between the activities of neighboring neurons are observed ubiquitously across systems and species and are dynamically regulated by several factors such as the stimulus' spatiotemporal extent as well as by the brain's internal state. Using the electrosensory system of gymnotiform weakly electric fish, we recorded the activities of pyramidal cell pairs within the electrosensory lateral line lobe (ELL) under spatially localized and diffuse stimulation. We found that both signal and noise correlations were markedly reduced (>40%) under the latter stimulation. Through a network model incorporating key anatomical features of the ELL, we reveal how activation of diffuse parallel fiber feedback from granule cells by spatially diffuse stimulation can explain both the reduction in signal as well as the reduction in noise correlations seen experimentally through independent mechanisms. First, we show that burst-timing dependent plasticity, which leads to a negative image of the stimulus and thereby reduces single neuron responses, decreases signal but not noise correlations. Second, we show trial-to-trial variability in the responses of single granule cells to sensory input reduces noise but not signal correlations. Thus, our model predicts that the same feedback pathway can simultaneously reduce both signal and noise correlations through independent mechanisms. To test this prediction experimentally, we pharmacologically inactivated parallel fiber feedback onto ELL pyramidal cells. In agreement with modeling predictions, we found that inactivation increased both signal and noise correlations but that there was no significant relationship between magnitude of the increase in signal correlations and the magnitude of the increase in noise correlations. The mechanisms reported in this study are expected to be generally applicable to the cerebellum as well as other cerebellum-like structures. We further discuss the implications of such decorrelation on the neural coding strategies used by the electrosensory and by other systems to process natural stimuli.

## Author Summary

Correlated activity is observed ubiquitously in the CNS but how activation of specific neural circuits affects correlated activity under behaviorally relevant contexts is poorly understood. Here, through a combination of electrophysiology, pharmacology, and mathematical modeling, we show that activation of the same parallel fiber feedback pathway leads to simultaneous reductions in both signal and noise correlations via independent mechanisms. Specifically, we show that feedback in the form of a negative image of the stimulus is necessary in order to attenuate signal but not noise correlations. Moreover, we show that trial-to-trial variability in the spiking responses of neurons providing this feedback is necessary to attenuate noise but not signal correlations. Our model thus predicts that activation of the same feedback pathway can simultaneously reduce both signal and noise correlations through independent mechanisms. In agreement with modeling prediction, pharmacological inactivation led to a strong increase in both signal and noise correlations but the magnitude of the change in signal correlation was not related to the magnitude of the change in noise correlations. Our proposed mechanism for simultaneous control of both signal and noise correlations is generic and is thus likely to be applicable to the cerebellum and to other cerebellar-like structures.

## Introduction

Understanding how the brain processes sensory information in order to lead to perception and behavior remains a central problem in neuroscience. Mounting evidence suggests that studying correlations between neurons is required to understand the neural code [1]–[4]. Such correlations have been observed across systems and species and can have profound impact on neural population coding by, e.g., either decreasing or increasing information transmission depending on their sign [2], [3], [5]–[7]. Experimental results have further shown that correlations between neurons are not static but are instead dynamically regulated by the spatiotemporal structure of sensory input [8]–[10] as well as higher order cognitive processes such as attention [11], [12]. In particular, attentional processes can reduce correlations between neural responses [11], which is thought to reduce redundancy and thus maximize information transmission as originally proposed by Barlow [13], [14]. Theoretical studies have proposed cellular and circuit mechanisms that can modulate correlated activity [15]–[18] but it is at best unclear how applicable these are in general [7].

Wave-type gymnotiform weakly electric fish offer an attractive model system for studying modulation of correlated activity because of well-characterized anatomy and natural sensory stimuli [19]–[21]. These fish actively generate an electric field around their body through the electric organ discharge (EOD). They can sense perturbations of this field caused by objects with conductivity different than that of the surrounding water (e.g. prey, conspecifics) through an array of electroreceptors on their skin surface that synapse onto pyramidal cells within the electrosensory lateral line lobe (ELL). Anatomical and physiological studies have shown large heterogeneities within the pyramidal cell population: on one hand, superficial pyramidal cells (SPs) have large apical dendritic trees and receive large amounts of plastic indirect feedback via parallel fibers [22], [23] while, on the other hand, deep pyramidal cells (DPs) instead have small apical dendrites and are thought to receive little or no indirect feedback [22], which is supported by results showing that pharmacological inactivation of indirect feedback input has a strong effect on SPs and little or no effect on DPs [24], [25]. While all pyramidal cells project to the midbrain Torus semicircularis and higher brain areas, only DPs project to the Eminentia Granularis posterior (EGp) and give rise to the feedback input to SPs [22].

Multiunit recording from ELL pyramidal cells have revealed that the baseline activities of neighboring pyramidal cells are significantly correlated [26], [27]. Further, such correlated activity is highly modulated based on the spatial extent of the stimulus. Indeed, stimuli mimicking prey items whose spatial extent was constrained to a small portion of the sensory epithelium (i.e. local) induced stronger correlations than stimuli mimicking conspecifics whose spatial extent was commensurate with the sensory epithelium (i.e. global) [26]. Interestingly, this effect was observed for both signal (i.e. correlations due to the fact that the neurons receive a common stimulus) and noise (i.e. correlations between the trial-to-trial variabilities of neurons) correlations. Pyramidal cells receive large amounts of feedback including a diffuse pathway consisting of parallel fibers from granule cells within the EGp [28], thereby making the ELL a cerebellar-like structure [29]. One of the functions of this feedback pathway, which is activated by global but not local stimulation [22], [24], [25], [30], is to attenuate the responses of single SPs but not DPs to low temporal frequency (i.e. <15 Hz) global stimulation relative to local stimulation by providing a negative image that “cancels out” the stimulus: thereby decorrelating the stimulus and the single neuron response [22], [24], [25], [30]–[34]. Recent studies have shown that a burst-time dependent plasticity observed experimentally is necessary to obtain this negative image at the single neuron level and to observe such decorrelation [32], [33], [35]. At the population level, experimental and theoretical work suggests that this feedback pathway can contribute to reducing signal correlations amongst ELL pyramidal cells but did not include the burst-time dependent plasticity [26], [27]. Thus, how indirect feedback onto ELL pyramidal cells in the form of a negative stimulus image produced by burst-time dependent plasticity actually reduces signal correlations between the activities of ELL pyramidal cells has not been investigated to date. Moreover, the mechanisms that underlie the experimentally observed changes in noise correlations remain unknown.

In order to make progress towards understanding the mechanisms that reduce both signal and noise correlations in ELL pyramidal cells under global stimulation, we built a network model of the ELL based on recently published results that accurately reproduces the effects of feedback on single pyramidal cell activity [32], [33] and that incorporates spiking activity from individual granule cells. We systematically varied model parameters in order to understand the regimes in which activation of granule cell input can reduce both signal and noise correlations. We found that the formation of a negative image by burst-timing dependent plasticity led to a reduction in signal correlations but not noise correlations. On the other hand, including trial-to-trial variability in the spiking responses of granule cells reduced noise but not signal correlations. Thus, our model made two important predictions: 1) that activation of the same feedback input onto ELL pyramidal cells can simultaneously reduce both signal and noise correlations and; 2) that the reduction in signal and noise correlations are mediated by independent mechanisms. We next validated these predictions experimentally by pharmacologically inactivating parallel fiber input onto ELL pyramidal cells. Consistent with prediction 1), we observed an increase in both signal and noise correlations after inactivation and; consistent with prediction 2), the increase in signal correlations was not related to the increase in noise correlations. Our combined experimental and modeling results thus provide a generic mechanisms by which parallel fiber feedback originating from cerebellar granule cells can simultaneously reduce both signal and noise correlations that are likely to be generally applicable across cerebellum and cerebellar-like structures in the brain.

## Results

### Model description

**Figure 1:**
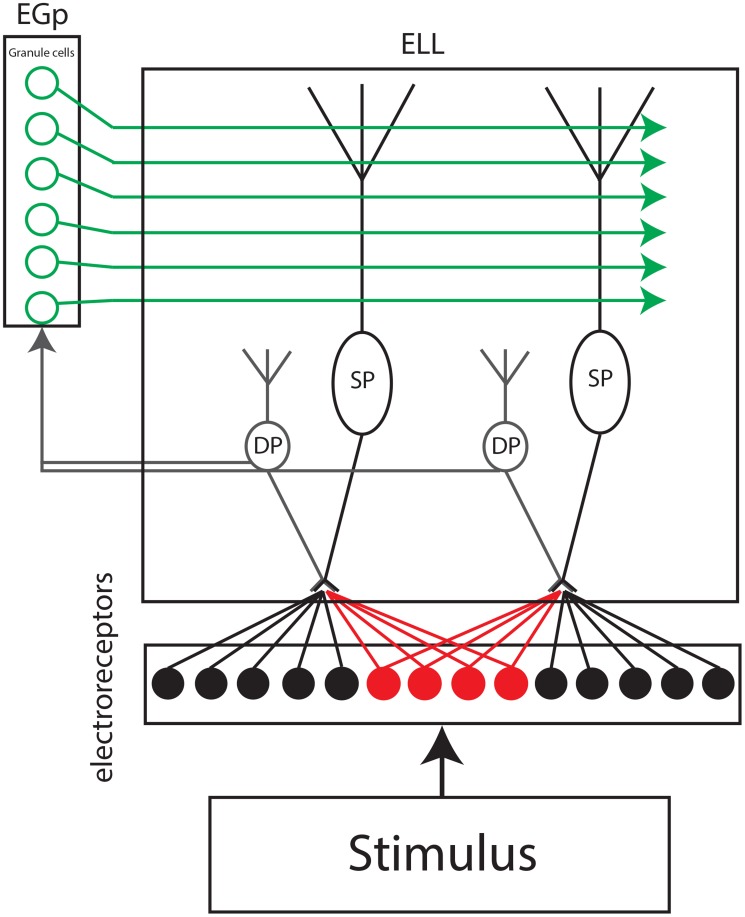
Anatomical and model schematic. Peripheral electroreceptor afferents receive the sinusoidal stimulus and project to both deep pyramidal (DP) as well as superficial pyramidal (SP) cells. The DPs relay the stimulus faithfully to a set of granule cells within the Eminentia Granularis posterior (EGp) that make direct excitatory synaptic contact onto SPs via parallel fibers as well as indirect inhibitory synaptic contact via local interneurons. It is assumed that each granule cell responds to a given phase of the sinusoidal stimulus and project via excitation and inhibition to SPs.

Our model is described in Fig. 1. The electroreceptor afferent population activity is assumed to faithfully follow the timecourse of the stimulus as observed experimentally [19], [36] (note that electroreceptor afferent spiking activities do not display significant noise correlations [37]) and synapse onto SPs. Since experimental studies have shown that noise correlations were proportional to the amount of receptive field overlap between pyramidal cells [26], which is largely due to feedforward input from electroreceptor afferents [23], we assumed that noise correlations between our model SPs were due to the fact that they receive shared input from electroreceptor afferents and were modeled using shared noise with √c being the fraction of common feedforward noise input (see Methods). DPs are also assumed to faithfully follow the timecourse of the stimulus as seen experimentally [24], [25] and relay this information to granule cells within the EGp. As seen experimentally, we assume that granule cells phase lock in response to sinusoidal global stimulation and are otherwise silent in the absence of stimulation as well as during local stimulation [22], [24], [25], [38]–[40]. Further, as assumed in previous modeling studies [32], [33], only a few granule cells are active at a given phase of the sinusoidal stimulus. However, unlike these, we implemented spiking mechanisms for each granule cell, considered trial-to-trial variability in their responses to the stimulus, and moreover considered the effects of granule cell input onto ELL pyramidal cells on correlated activity at the populations level (see Methods).

### Correlations between pyramidal cell spiking activities are stimulus-dependent

**Figure 2:**
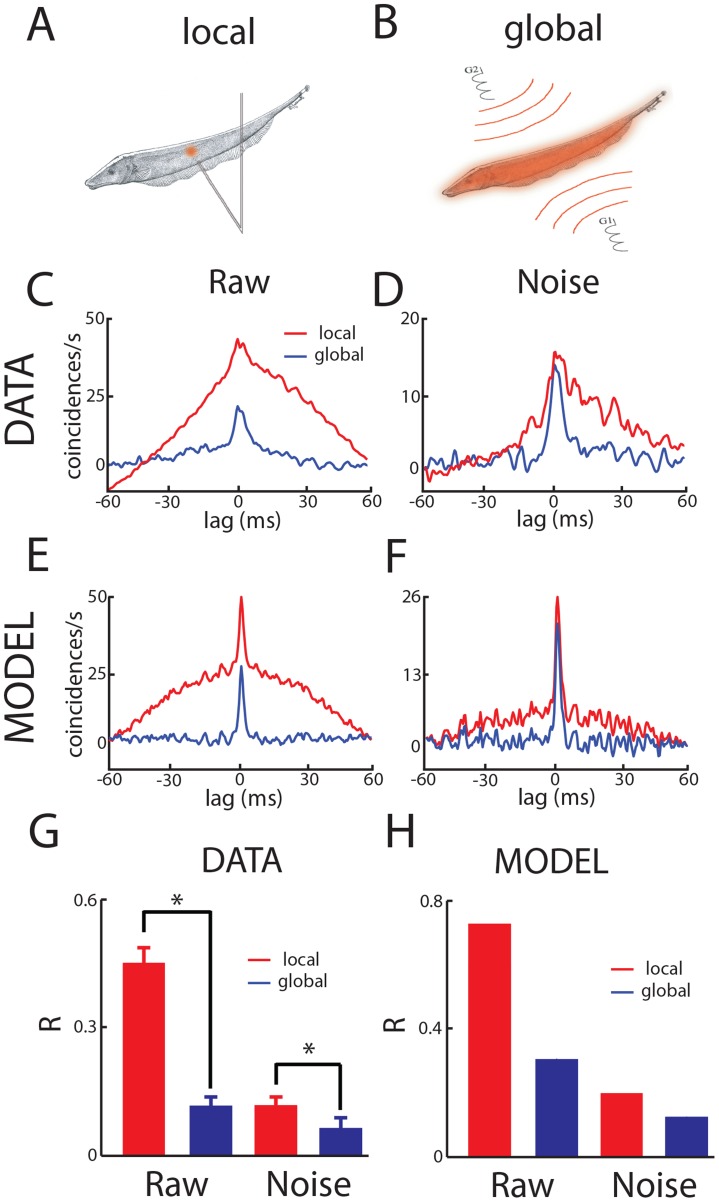
Stimulation geometry strongly influences signal and noise correlations in ELL pyramidal cells. **A**) For local stimulation geometry, the stimulus is delivered via a small dipole located close to the animal's skin. **B**) For global stimulation, the stimulus is instead delivered via two electrodes located 15 cm away from the animal on each side. **C**) Population-averaged cross-correlograms (CCGs) from same type pyramidal cell pairs recorded from simultaneously for global (blue) and local (red) 4 Hz sinusoidal stimulation. **D**) Population-averaged noise CCGs from same type pyramidal cell pairs recorded from simultaneously for global (blue) and local (red) 4 Hz sinusoidal stimulation. **E**) CCGs from our model under simulated global (blue) and local (red) 4 Hz sinusoidal stimulation. **F**) Noise CCGs from our model under simulated global (blue) and local (red) 4 Hz sinusoidal stimulation. **G**) Population-averaged cross-correlation coefficient R for global (blue) and local (red) 4 Hz sinusoidal stimulation. **H**) Population-averaged cross-correlation coefficient R for our model under simulated global (blue) and local (red) 4 Hz sinusoidal stimulation. “*” indicates statistical significance at the p = 0.05 level using a signrank test with N = 18.

We recorded from pyramidal cell pairs (see Methods) in response to sinusoidal stimuli with frequency 4 Hz that were delivered either locally (Fig. 2A) via a small dipole positioned lateral to the animal or globally (Fig. 2B) via two electrodes positioned far from the animal on each side. These stimuli were used because of their relative simplicity, behavioral relevance, and because previous studies have shown that globally but not locally given 4 Hz sinusoidal stimuli will activate feedback from the EGp onto ELL pyramidal cells [22]. It is important to realize that the temporal aspect of the stimulus is the same in both cases and that it is only the spatial extent that increases as we go from local to global stimulation. Correlations between the recorded spike trains were quantified using the cross-correlogram (CCG) that gives the number of coincident spikes per unit time relative to chance levels as a function of lag. The CCGs obtained under local and global 4 Hz sinusoidal stimulation were mostly symmetric with respect to the 0 lag, indicating that correlated activity most likely arises because of common input (Fig. 2C). However, we found marked differences in their structure that were contingent on the stimulus' spatial extent. Indeed, despite the fact that global stimulation impinges on most if not all of the sensory epithelium and might thus be expected to increase correlated activity, the CCG obtained under local stimulation was actually larger than that obtained under global stimulation (Fig. 2C, compare red and blue lines). To separate the contributions of signal and noise correlations to the CCG, we used the shuffle predictor [41] and computed the noise CCG. The noise CCG obtained under local stimulation was also significantly larger than that obtained under global stimulation (Fig. 2D, compare red and blue lines).

Previous studies have shown that activation of granule cell input onto ELL pyramidal cells under global stimulation can reduce single neuron responses to stimulus by forming a negative image of the stimulus [22], [24], [34], which can attenuate signal correlations at the network level [27]. The formation of this negative image is mediated by anti-Hebbian burst-time dependent plasticity at parallel fiber-ELL synapses [32], [33], [35]. In order to gain better understanding as to how activation of parallel fiber input onto ELL pyramidal cells could mediate the experimentally observed changes in correlated activity, we used the model described in Fig. 1 that includes the anti-Hebbian burst-time dependent plasticity (see Methods) in order to mediate the formation of a negative image. We found that, for suitable parameters, our model was able to reproduce the experimentally observed changes in both the raw (Fig. 2E) and the noise CCG (Fig. 2F). The magnitude of correlation was assessed using the cross-correlation coefficient (see Methods). Overall, the cross-correlation coefficient as well as the noise cross-correlation coefficient were reduced by >40% when we transition from local to global stimulation in the data (Fig. 2G). We found that our model was able to successfully reproduce these results (Fig. 2H).

### Anti-Hebbian burst timing dependent plasticity allows for a reduction in signal correlations

**Figure 3:**
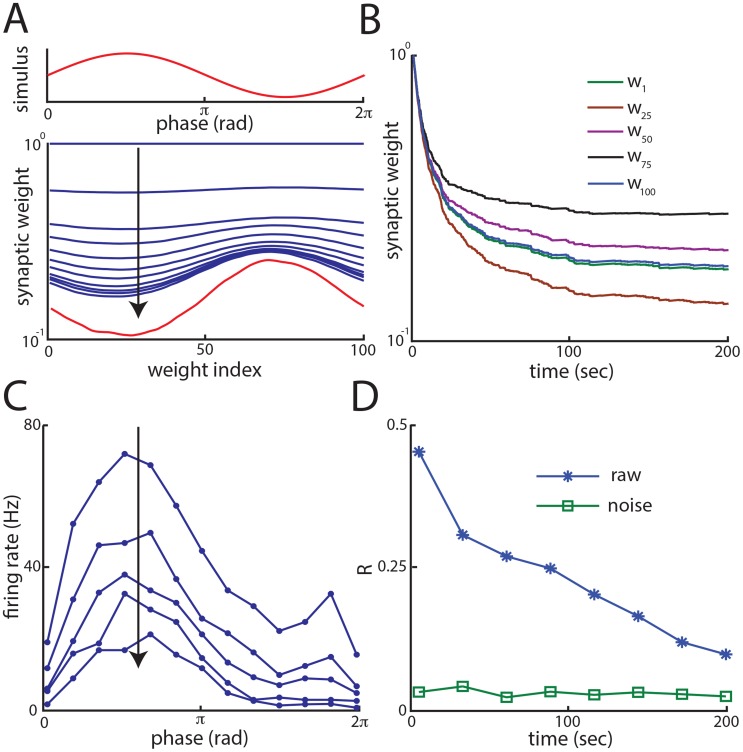
The formation of a negative image mediated by anti-Hebbian burst time dependent plasticity reduces signal but not noise correlations. **A**) One cycle of the 4 Hz sinusoidal stimulus (top, red trace) and synaptic weights (bottom, blue traces) as a function of time for (black traces from top to bottom), t = 0, t = 0.5 sec, t = 1 sec, t = 1.5 sec, t = 2 sec, t = 2.5 sec, t = 3 sec, t = 3.5 sec, t = 4 sec, t = 4.5 sec, t = 5 sec, t = 5.5 sec. The red trace (bottom) shows the synaptic weights at t = 1000 sec (steady state) for comparison. The vertical arrow shows the progression of time. **B**) Time series of 5 synaptic weights during training. **C**) Cycle histograms from one SP cell neuron for the synaptic weights corresponding to (from top to bottom): t = 25 sec, 50 sec, 75 sec, 100 sec, 150 sec, and 200 sec. The vertical arrow shows the progression of time. Note the progressive reduction in response modulation as the negative image forms. **D**) Raw and noise correlation computed for the synaptic weights values obtained during training for the same time shown on the x-axis. Note the progressive decrease in signal correlations but the relative constancy of noise correlations.

Why does our model successfully reproduce the experimental data? We first investigated the effects of burst-timing dependent plasticity. Consistent with previous results [32], the synaptic weights settled to their equilibrium values over time to form a negative image of the stimulus over time (Figs. 3A, 3B): weights of granule cells that fire at the stimulus' local maximum were depressed the most while those of granule cells that fire at the stimulus' local minimum were depressed at least (Fig. 3A, compare red traces). As expected, the progressive formation of the negative image progressively reduced the variations in the single model SP cell's firing rate due to the stimulus (Fig. 3C), which is consistent with previous results [32] and experimental data [22]. At the population level, our results show that the formation of the negative image reduces the overall correlations between model SP cells over time (Fig. 3D). However, as we did not observe any changes in noise correlations (Fig. 3D), we conclude that it is signal correlations only that are attenuated. Therefore, our model shows that the mechanisms that lead to the formation of a negative image are sufficient to explain the overall reduction in signal correlations observed when parallel fiber feedback input onto ELL pyramidal cells is activated. However, as no change in noise correlation was observed, we conclude that these are regulated by other mechanisms.

### Trial-to-trial variability in the spiking activity of granule cells is necessary in order to reduce noise correlations in ELL pyramidal cells

**Figure 4:**
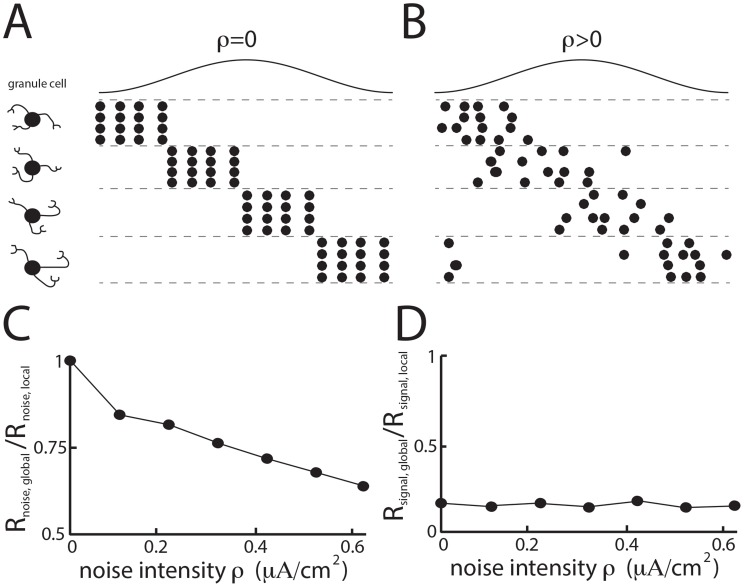
Trial-to-trial variability of granule cell firing reduces noise correlations under global stimulation. **A**) Raster plots showing responses of four different granule cells to repeated cycles of the sinusoidal stimulus in the deterministic regime for the granule cells (i.e. *ρ* = 0). **B**) Raster plots showing trial-to-trial variability in the responses of four different granule cells to repeated cycles of the sinusoidal stimulus in the stochastic regime for the granule cells (i.e. *ρ*>0). **C**) Ratio of the noise correlation coefficients obtained under simulated local and global stimulation as a function of the noise intensity *ρ*. Note that the ratio decreases from 1 as *ρ* is increased from 0. **D**) Ratio of the signal correlation coefficients obtained under simulated local and global stimulation as a function of the noise intensity *ρ*.

In order to gain understanding as to the mechanisms by which activation of granule cell input onto ELL pyramidal cells reduces noise correlations in our model, we plotted the spiking activities of four example model granule cells each firing at a different phase of the sinusoidal input in the deterministic regime (i.e. no intrinsic noise) and in the stochastic regime (i.e. intrinsic noise). In the former regime, there is no trial-to-trial variability in the spiking activity of a given granule cell (Fig. 4A) while this is not the case in the latter regime (Fig. 4B) (we note that previous studies did not consider spiking activity of granule cells or trial-to-trial variability [32], [33]). We found that increasing the noise intensity ρ in the granule cells led to a decrease in the noise correlation coefficient under global stimulation relative to that obtained under local stimulation (Fig. 4C). Importantly, the noise correlation coefficients obtained under local and global stimulation were equal in the absence of noise (i.e. ρ = 0). In contrast, increasing the noise intensity ρ did not affect the signal correlation coefficient obtained under global stimulation relative to that obtained under local stimulation (Fig. 4D). Thus, trial-to-trial variability in the granule cell spiking activity is not necessary in order to observe a reduction in the amount of signal correlations, which is consistent with previous studies [27] and is discussed further below. However, our results reveal that trial-to-trial variability in the spiking activities of granule cells is necessary in order to observe reduced noise correlations under global stimulation. Thus, our model shows that the mechanism that mediates the reduction in noise correlations (i.e. trial-to-trial variability in granule cell spiking activity) is independent of the one that mediates the reduction in signal correlations (i.e. the negative image that is formed because of anti-Hebbian burst-timing dependent plasticity).

### Reduction in noise correlation is robust to changes in model parameters

We next systematically varied model parameters in order to test whether the changes in noise correlation were robust. We first varied the correlation coefficient between the noise sources to each SP cell *c* as well as the correlation coefficient *e* between the noise sources to each granule cell and the DP cell population. Thus, intuitively, *c* represents the correlation coefficient between the variabilities in peripheral receptor afferent activities projecting to each SP cell, which of course increases with increasing fraction of shared receptor afferent input. On the other hand, *e* represents the correlation coefficient between the noise received by the granule cells and that received by the DP cells. Thus, *e* will be high if most of the variability displayed by granule cells is inherited from the input that they receive from DP cells and low if most of the variability is due to intrinsic mechanisms (i.e. random openings of ion channels). We note that we assumed that the sources of noise to the DP and SP cells within a given ELL column were the same, which is consistent with anatomical findings showing almost complete overlap from sources of feedforward input [23] (see Methods).

**Figure 5:**
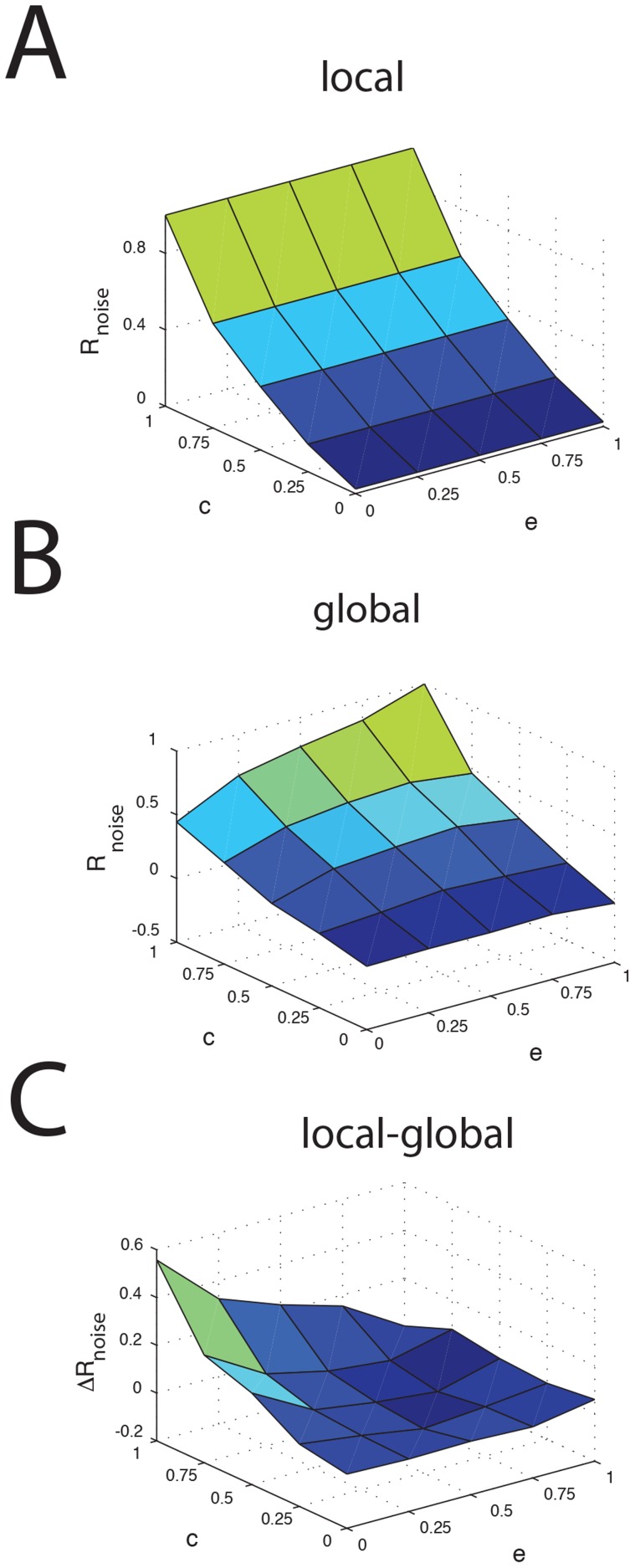
Correlated variability between SPs and granule cells influences noise correlations. **A**) Noise correlation coefficient as a function of the input noise correlation coefficient *c* for the SP cells and of the fraction of shared noise with the SPs *e* for local stimulation. **B**) Noise correlation coefficient as a function of *c* and *e* for global stimulation. **C**) Reduction (local-global) in noise correlation coefficient as a function of *c* and *e*.

Under simulated local stimulation (i.e. no feedback), the noise correlation coefficient is solely determined by the amount of shared noise *c* received by both SPs (Fig. 5A). Thus, we have R_noise,local_ = 1 when c = 1 and R_noise,local_ = 0 when c = 0. Since we assume that the granule cell population is silent during local stimulation, the fraction of shared noise with the SPs *e* has no effect on R_noise,local_. Under simulated global stimulation, for which the granule cells are active, we observed a reduction in noise correlation as both *c* and *e* tend towards zero (Fig. 5B). Plotting the difference between the noise correlation coefficients obtained under local and global stimulation revealed that the greatest reduction in noise correlations was seen for *c* near unity and *e* close to zero (Fig. 5C). Thus, our model predicts that, for a given intensity, trial-to-trial variability in the granule cell spiking activity is most effective at reducing noise correlations amongst SPs when the trial-to-trial variabilities of SP cells are strongly positively correlated in the first place and when trial-to-trial variability in granule cell firing activity is weakly correlated with trial-to-trial variability in the DP cell population. Intuitively, this makes sense as the variability of the granule cell population is then largely independent of the variability in the SP cells, which will reduces noise correlations between the spiking activities of SP cells.

**Figure 6:**
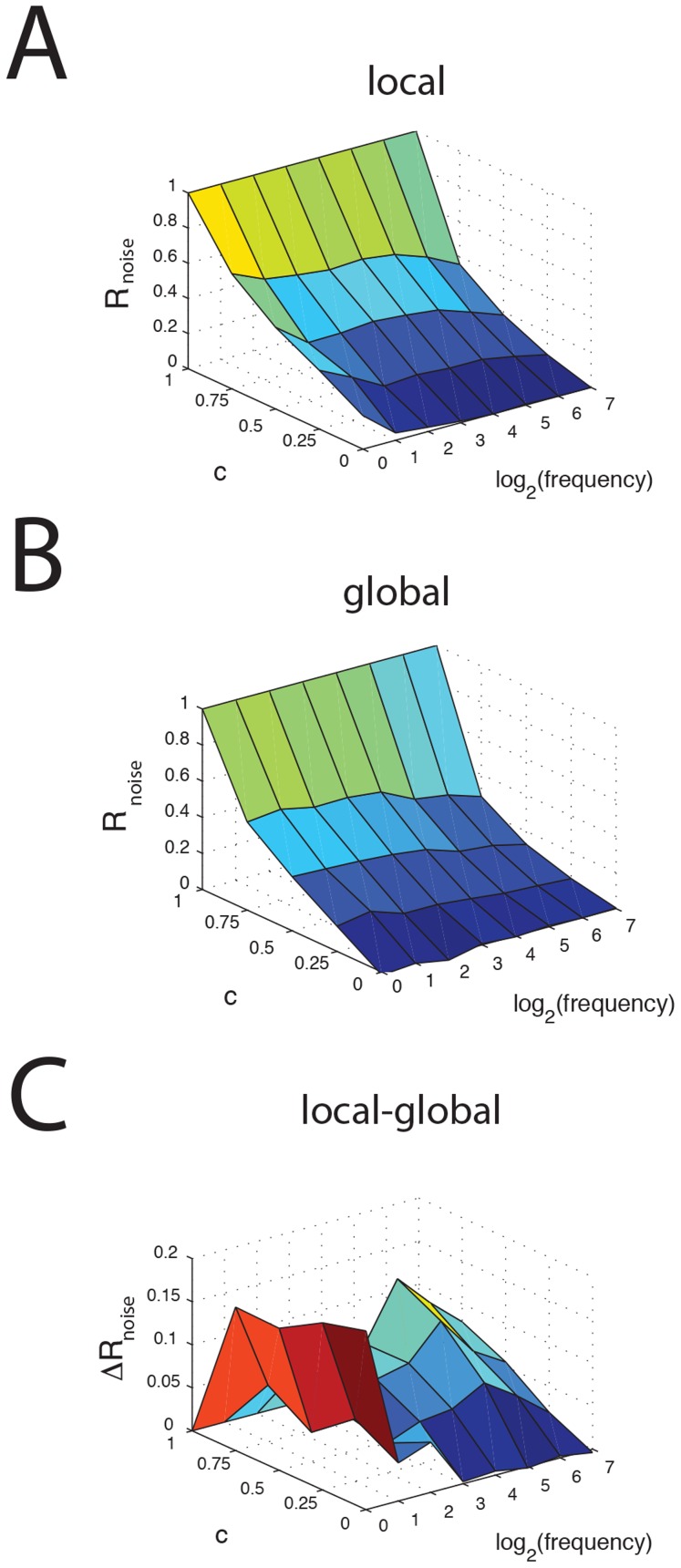
Reduction of noise correlations by granule cell activity is dependent on stimulation frequency. **A**) Noise correlation coefficient as a function of *c* and the sinusoidal stimulation frequency *f* for local stimulation. **B**) Noise correlation coefficient as a function of *c* and *f* for global stimulation. **C**) Reduction (local-global) in noise correlation coefficient as a function of *c* and *f*.

We next systematically varied both the fraction of shared noise received by the SPs *c* as well as the stimulation frequency *f*. For simulated local stimulation (i.e. no feedback), the noise correlation coefficient R_noise,local_ had a similar dependence on *c* independently of stimulation frequency *f* (Fig. 6A). However, under simulated global stimulation (i.e. with feedback), the noise correlation coefficient R_noise,global_ decreased as a function of stimulation frequency *f* for a given value of c except for *c* = 1 and *c = 0* (Fig. 6B). As such, plotting the difference between R_noise,local_ and R_noise,global_ reveals that the reduction in noise correlation tends to be greatest for low stimulation frequencies (i.e. <16 Hz) as well as when SPs receive large amounts of correlated input (Fig. 6C). Thus, our model predicts that the reduction in noise correlation will be greatest for low stimulation frequencies and for pyramidal cell pairs with high fraction of shared input (i.e. large amounts of receptive field overlap). Intuitively, this makes sense as the tendency for pyramidal cells to display burst firing is greatest for low frequency stimulation [32], [42] and because burst firing in ELL pyramidal cells tends to increase correlations between their spiking activities [26].

### Pharmacological inactivation of parallel fiber feedback onto ELL pyramidal cells shows that the same feedback pathway simultaneously reduces both signal and noise correlations via independent mechanisms

**Figure 7:**
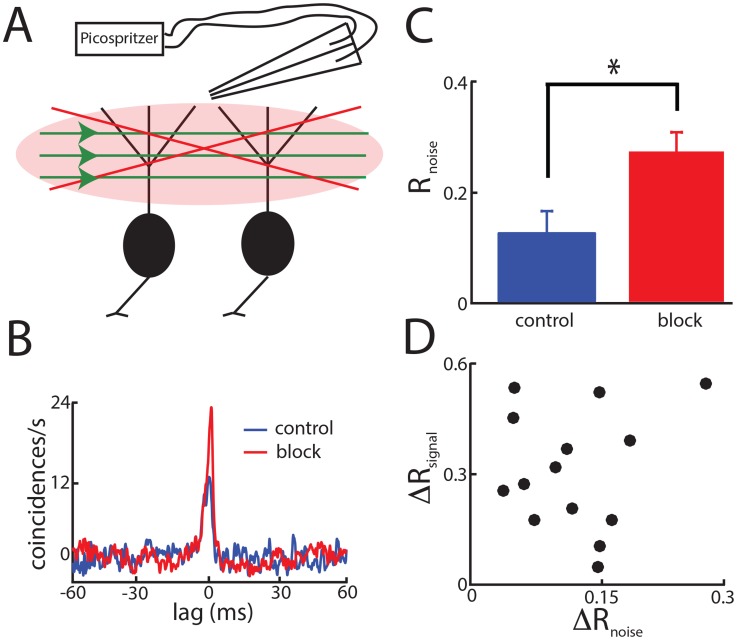
Pharmacological inactivation of indirect feedback input onto ELL pyramidal cells increases noise correlations. **A**) Schematic of the pharmacological inactivation technique. **B**) Noise CCG from an example pair of same type ELL pyramidal cells under 4 Hz global stimulation before (control, blue) and after (block, red) pharmacological inactivation of feedback pathways. **C**) Population-averaged noise correlation coefficients under control (blue) and during the block (red). “*” indicates statistical significance at the p = 0.05 level using a signrank test with N = 14. **D**) Change in signal correlation coefficient (block-control) as a function of the change in noise correlation coefficient (block-control). No significant correlation was observed between both quantities (R = 0.32, p = 0.18, N = 14).

Our model made two important predictions: 1) that the activation of the same feedback pathway simultaneously reduces both signal and noise correlations and; 2) that it does so via independent mechanisms. We tested these predictions experimentally by pharmacologically inactivating parallel fiber input onto ELL pyramidal cells (Fig. 7A, see Methods). Consistent with modeling prediction 1), pharmacological inactivation under global stimulation led to a significant increase in both signal and noise correlations (p<0.05, signrank tests) (Figs. 7B,C,D). We note in passing that previous studies have shown that pharmacological inactivation under local stimulation did not have such noticeable effects [22], [24], [25]. Moreover, to test prediction 2), we plotted the increase in signal correlation as a function of the increase in noise correlations (Fig. 7D). If our modeling prediction were correct, then we would expect to see no significant relationship between both quantities. Consistent with our modeling prediction, no significant correlation was observed (R = 0.32, p = 0.18, N = 14).

## Discussion

### Summary of results

In this study, we presented new experimental results showing that correlated activity in ELL pyramidal cells is reduced under global 4 Hz sinusoidal stimulation relative to local 4 Hz sinusoidal stimulation. We observed significant reduction in both signal and noise correlations as was observed previously using broadband (0–120 Hz) noise stimulation [26], [27]. In order to explain the observed reduction in noise correlations, we extended a previously published model that accurately describes how feedback can attenuate the responses of single pyramidal cells to global stimulation [32], [33] to include: 1) correlated activity of multiple pyramidal cells and; 2) trial-to-trial variability in the spiking activities of individual granule cells. Simulation of this model revealed that it could qualitatively explain the changes in both the signal and noise correlations observed experimentally. The formation of a negative image of the stimulus via anti-Hebbian burst timing dependent plasticity was necessary to induce a reduction in signal but not noise correlations. In contrast, the magnitude of trial-to-trial variability in granule cell spiking activity strongly influenced the magnitude by which noise but not signal correlations are reduced under global stimulation. By systematically varying model parameters, we found that this reduction was strongest in our model when the shared noise amongst pyramidal cells was highest, when the noise sources between the pyramidal and the granule cell populations were least correlated, and for low sinusoidal stimulation frequencies. Our model made the important predictions that activation of the same feedback pathway can simultaneously reduce both signal and noise correlations via independent mechanisms. We verified these predictions by pharmacologically inactivating parallel fiber feedback input onto ELL pyramidal cells. Consistent with modeling predictions, pharmacological inactivation led to increases in both signal and noise correlations that were not significantly related to one another.

### Reduced noise correlations under global stimulation, implications for neural coding

The effects of noise correlations on neural coding have been the subject of much study and it is generally agreed that noise correlations will limit information transmission by introducing redundancy in the neural code [2], [3], [43]–[46] (but see [47]). In particular, even small amounts of noise correlations can have significant effects on information transmission by large neural populations [45]. From this point of view, if the brain is trying to maximize information transmission, then redundancy must be minimized by reducing noise correlations [14]. While the reduced noise correlations observed under some behavioral states [11], [48] does lend some support to this hypothesis, the fact that neural correlations are dynamically regulated across systems and species instead suggest that the brain uses different coding strategies based on both its internal state as well as the spatiotemporal characteristics of the stimulus [7], [13].

In wave-type gymnotiform weakly electric fish, local and global stimuli arise in different behavioral contexts: local stimuli are mostly caused by prey [49] while global stimuli are caused in part by conspecifics [50], [51]. The fact that single pyramidal neurons display reduced responses to low frequency global stimuli [22], [24], [25], [31], [52] is thought to better enable them to detect signals caused by prey items [27]. At the population-level, we propose that activation of parallel fiber feedback input onto ELL pyramidal cells by low frequency global stimuli contributes to reducing noise correlations in the superficial pyramidal cell population. This would enable downstream neurons within the midbrain Torus semicircularis to better detect transient increases in correlated activity that would be caused either by prey stimuli as well as communication stimuli. Indeed, previous studies have shown that single ELL pyramidal cells will increase the precision of their spike timing in response to chirp stimuli which is expected to give rise to increase in correlated activity at the population level [53], [54]. While additional support for this hypothesis comes from evidence showing that neurons within the torus semicircularis can respond selectively to communication stimuli [55], [56] (see [20] for review), further studies investigating how ELL pyramidal cell populations respond to communication stimuli are needed in order to test these predictions.

Previous studies have shown that there are large heterogeneities in the pyramidal cell population: while SPs receive the largest amount of feedback, DPs instead receive little or no feedback [23], [57]. Previous studies point to different functional roles for these different classes as DPs actually give rise to the feedback input to SPs [22]. All evidence shows that the activities of single DPs is not dependent on the stimulus' spatial extent and that these cells tend to be broadly tuned [24], [25], [58]. At the population-level, correlations between DPs are also largely independent of the stimulus' spatial extent [27]. All these results support the hypothesis that deep pyramidal cells give a faithful representation of the incoming sensory input that is: 1) sent to higher brain centers and 2) used to reduce single SP cell responses as well as decorrelated SP cell activity at the population level to low frequency global stimuli.

### Implications for other systems

Our results showing that activation of granule cell spiking activity by spatially diffuse stimuli can reduce noise correlations are likely to be applicable to other systems. This is because the ELL shares many anatomical features with the mammalian cerebellum as well as other cerebellar-like structures such as the dorsal cochlear nucleus for which a layer of principal cells receive parallel fiber input from a set of granule cells [59]. In fact, our proposed mechanisms by which activation of granule cell input onto ELL pyramidal cells actually reduces correlated activity are likely to be applicable to other cerebellum-like structures as well as the cerebellum. In fact, they might help explain seemingly paradoxical experimental results showing that correlations between the simple activities of pairs of cerebellar Purkinje cells is low even when they receive common parallel fiber input [60].

We note that our modeling assumption that granule cells fire at all phases of the input is reasonable given available anatomical data [61]. The trial-to-trial variability in granule cell firing responses to the stimulus is also consistent with experimental data from mormyrid weakly electric fish [62]. While the source of this trial-to-trial variability is still a matter of debate, the fact that granule cells are compact and thus receive only a small number of inputs makes it likely that this variability is the result of both the random openings of ion channels (intrinsic) as well as inherited from input from the DP population. Our modeling results show that the relative amount of noise shared with pyramidal cells does not qualitatively influence the overall reduction of noise correlations over a wide range.

Recent results from a recent study in mormyrid weakly electric fish have shown that, while granule cells are activated with a wide distribution of delays, the distribution was not uniform [62], which is unlike the assumption made here. However, we do not expect this to be an issue as long as there is at least one granule cell that is firing at any given phase of the sinusoidal input whose trial-to-trial variability will effectively increase the amount of uncorrelated noise received by the pyramidal cell population and thereby reduce noise correlations.

Finally, we note that there exists important similarities between the electrosensory and visual systems. Indeed, like primary visual cortical (i.e. V1) neurons [63], ELL pyramidal neurons respond to stimulation within a particular region of sensory space (i.e. the classical receptive field) [31]. Importantly, responses to classical receptive field stimulation are modulated by stimulation outside but within the non-classical receptive field in similar ways in both systems. Specifically, non-classical receptive field stimulation decorrelates the single neural response from low frequency as well as enhances information transmission about higher frequency classical receptive field stimulation [52], [64], [65]. Modeling studies in the visual system have proposed that such decorrelation is a form of predictive coding that removes the redundant aspects of natural visual stimuli [66]–[68]. We note that this is also the case for ELL pyramidal cells [22], [27]. Previous studies have shown that, for ELL pyramidal cells, at least part of the non-classical receptive field effects are being mediated by indirect feedback [24]. In the visual system, both anatomical [69] and modeling [66] studies support the hypothesis that the non-classical receptive field of V1 neurons also originates, at least in part, from feedback input. It is thus likely that the effects described here also apply to the neurons within the primary visual cortex, where activation of feedback input via non-classical receptive field stimulation also decorrelates neural responses to classical receptive field stimulation at the population level [64].

### Conclusion

We have shown a viable mechanism by which sensory neuron populations can have correlations within their trial-to-trial variabilities markedly reduced under a particular behavioral context. This mechanism is expected to be generally applicable to cerebellum and other cerebellar-like structures. Further experimental studies recording from cerebellar granule cells in gymnotiform weakly electric fish are needed to verify our modeling predictions.

## Methods

### Ethics statement

McGill University's animal care committee approved all procedures.

### Animals and surgery

The weakly electric fish *Apteronotus leptorhynchus* was used exclusively in these studies. Fish were purchased from tropical fish suppliers and were acclimated to laboratory conditions and housed in groups of 8–10. Water conductivity was between 400 and 800 µS, the pH was maintained between 6.8 and 7.2, and the temperature was kept between 27 and 29°C [70], [71]. Surgical procedures were explained in detail previously [26], [72]–[74]. Briefly, to immobilize the fish, we injected 0.1–0.5 mg of tubocurarine (Sigma) intramuscularly. We then transferred the fish to a recording tank and respirated it via a mouth tube at a flow rate of 10 mL/min. We glued a metal post rostral to the exposed area of the skull after topical application of lidocaine (2%) to stabilize the head during recording. We then drilled a small hole of ∼2 mm^2^ over the cerebellum and the ELL area, caudal to the border between hindbrain and midbrain in order to access the pyramidal neurons [56], .

### Recordings

Extracellular recordings from pyramidal cells within the centro-lateral and lateral segments were obtained using metal filled micropipettes [77]. Recordings from N = 18 pyramidal cell pairs were achieved as described previously [26], [27]: two separate electrodes were advanced independently in order to ensure that a well-isolated single unit was present on each one. Recordings were sampled at 10 kHz and were digitized using a Power1401 with Spike2 software (Cambridge Electronic Design, Cambridge, UK). Previous studies have shown a strong negative correlation between the baseline firing rate (i.e. the firing rate in the presence of the animal's unmodulated EOD) and dendritic morphology [22], [57], such that deep pyramidal cells tend to have the highest (>30 Hz) firing rates while superficial pyramidal cells tend to have the lowest (<15 Hz) firing rates [24], [25], [31], [58], [72], [78].

### Pharmacology

Previous techniques were used to pharmacologically inactivate indirect feedback onto N = 9 pairs of ELL pyramidal cells [22], [24]–[27], [30], [74], [79]. Briefly, a double-barrel pipette was advanced into the ELL molecular layer. One barrel contained a glutamate solution (1 mM) while the other contained a solution of CNQX (1 mM), which is a non-NMDA glutamate receptor antagonist. All drugs were obtained from Sigma and were dissolved in saline. Both barrels were connected to a picospritzer (Hannifin). Pressure ejection of glutamate was used to determine whether the double-barrel pipette was in the vicinity of the cell pair recorded from: close vicinity typically resulted in short latency excitation of both cells [26]. We then ejected CNQX as done previously [24]–[26].

### Stimulation

As the electric organ discharge of *A. leptorhynchus* is neurogenic, it is not affected by immobilization with curare-like drugs. All stimuli consisted of amplitude modulations (AMs) of the animal's own EOD and were produced by applying a train of sinusoidal waveforms to the fish. Each sinusoid was triggered at the zero crossing of each EOD cycle and had a period slightly less than that of the EOD waveform; hence the train remains synchronized to the animal's discharge and, depending on its polarity, either adds to or subtracts from the animal's own discharge. A modulation waveform was then multiplied with the train of sinusoidal waveforms (MT3 multiplier; Tucker Davis Technologies) and the resulting signal was first isolated from ground (A395 linear stimulus isolator; World Precision Instruments) before being delivered using either global or local stimulation geometry. For global stimulation, signals were delivered through pairs of chloridized silver wire electrodes positioned 15 cm away from the fish in either side of the recording tank. In contrast, for local stimulation, we used a small local dipole electrode that was located 1–3 mm from the skin. The intensities of local and global stimuli were similar to those used previously [56], [74], [75] and were adjusted such as to give rise to similar changes in EOD amplitude as measured by a small dipole close to the animal's skin [22], [31]. Stimuli consisted of 4 Hz sinusoidal AMs of the animal's own EOD.

### Analysis

All analysis was performed using custom-built routines in Matlab (The Mathworks, Natick, MA). Action potential times were defined as the times at which the signal crossed a suitably chosen threshold value. From the spike time sequence we created a binary sequence X(t) with binwidth dt = 0.5 ms and set the content of each bin to equal the number of spikes the time of which fell within that bin. The auto-correlogram (ACG) *A(τ)* was computed using:
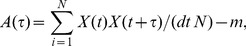
(1)where *N* is the total number of action potentials and *m* is the mean number of action potentials per unit time (i.e. the mean firing rate). The cross-correlogram (CCG) between spike trains *X_1_(t)* and *X_2_(t)*, *C(τ)*, was computed using:
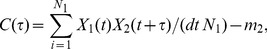
(2)where *N_1_* is the total number of action potentials for spike train *X_1_(t)* and *m_2_* is the number of action potentials per unit time for spike train *X_2_(t)*. We note that the sum is performed over the spikes of cell 1 and that the labeling of cells within the pair is completely arbitrary. The particular cell used for averaging does not matter for our data as the CCGs were symmetric with respect to lag 0 (see Fig. 2). The cross-correlation coefficient was computed for each cell pair as [44]:
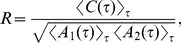
(3)where 
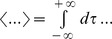
 denotes the average over lag *τ*.

We distinguished the contributions of signal and noise correlations to the CCG using the shuffle predictor [41], [80]. Let M be the total number of cycles of the sinusoidal stimulus and let *X_i,j_(t)* be the response of neuron i to cycle j at time t, the shuffle predictor is then a measure of signal correlations and is given by:
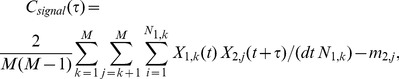
(4)where *N_1,k_* is the total number of action potentials for spike train *X_1,k_(t)* and *m_2,j_* is the number of action potentials per unit time for spike train *X_2,j_(t)*. The noise CCG, *C_noise_(τ)*, is then given by:
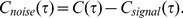
(5)


### Model

Our model is an extension of a model considered by previous studies [32], [33]. We consider two superficial ELL pyramidal cells (i.e. SP cells) from transmembrane voltages are solutions to the following system of equations:
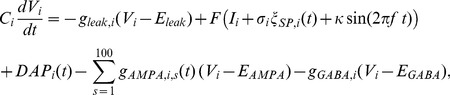
(6)where, for cell i, *V_i_* is the transmembrane voltage, *C_i_* is the membrane capacitance, *g_leak,i_* is the leak conductance, *I_i_* is a constant bias current, *σ_i_* is the noise standard deviation, *ξ_SP,i_* is low-pass filtered (fourth order Butterworth with cutoff frequency 500 Hz) Gaussian white noise with zero mean and variance unity, κ is the stimulus amplitude, *f* is the stimulation frequency, *DAP_i_(t)* is the depolarizing afterpotential current (described below), *g_GABA,i_* is a constant inhibitory conductance with reversal potential *E_GABA_*, and *g_AMPA,i,s_(t)* is the time varying excitatory conductance of parallel fiber *s* with reversal potential *E_AMPA_*. The function F(x) half-wave rectifies the input (i.e. F(x) = x for x>0 and F(x) = 0 otherwise).

The current *DAP_i_(t)* is given by [32], [33], [81]:
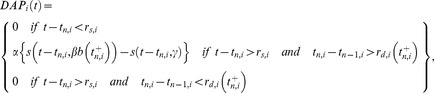
(7)where, for neuron *i*, *r_s,i_* is the absolute somatic refractory period, *r_d,i_(t)* is the absolute dendritic refractory period, *t_n.i_* is the last spike time, t_n-1,i_ is the next to last spike time, 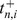 is the time immediately after *t_n.i_*, and s(t,a) is an alpha function given by:
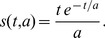
(8)The dendritic refractory period obeys the following system of equations:
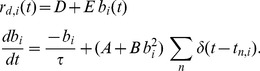
(9)


Each SP cell is modeled using integrate-and-fire formalism [82]. Thus, for cell *i*, when the voltage *V_i_(t)* reaches a threshold value *V_threshold,i_*, an action potential is said to have occurred and *V_i_(t)* is then immediately reset to the resting potential *V_rest,i_* and is maintained there for the duration of the absolute somatic refractory period *r_s,i_*. There are 100 AMPA synapses on each SP cell each emanating from one granule cell via a parallel fiber. Anatomical findings suggest that a single parallel fiber does indeed make synaptic contact with multiple pyramidal cells, but that the synaptic boutons are located far away from one another in the ELL [28]. It is thus likely that neighboring pyramidal cells receive synaptic input from largely disjoint sets of granule cells (Maler, Pers. Comm.). Thus, we assumed that each SP cell receives input from different sets of granule cells. The time varying post-synaptic conductance of AMPA synapse *s*, *g_AMPA,i,s_(t)*, obeys the following equation:
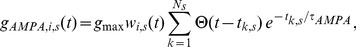
(10)where *g_max_* is the maximum conductance, *w_i,s_(t)* is the synaptic weight, *t_k,s_* is the *k* ^th^ spike of presynaptic granule cell *s*, *N_s_* is the total number of spikes fired by granule cell *s*, and Θ(x) is the Heaviside function (Θ(*x*) = 1 if x>0 and Θ(*x*) = 0 otherwise).

Each granule cell is modeled using an integrate-and-fire formalism similar to that used for the SP cell with the same threshold, reset, and absolute refractory period (note that we assume that separate sets of granule cells project to each pyramidal cell). For granule cell *i*, the transmembrane voltage obeys the following equation:
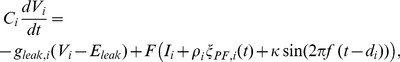
(11)where *ρ_i_* is the noise standard deviation, *ξ_PF,i_* is low-pass filtered (fourth order Butterworth with 500 Hz cutoff frequency), and *d_i_*, the delay for granule cell *i*, is given by:
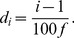
(12)We note that DPs are assumed to faithfully relay the sinusoidal stimulus to the granule cells, which is consistent with available experimental data [21], [31], [37], [58]. Each synaptic weight *w_i,s_* follows anti-Hebbian plasticity as described previously [32], [33]. Specifically, a previously described burst-dependent plasticity [35] learning rule reduces the value of a specific synaptic weight when pre and post-synaptic bursts of activity are coincident within a given time window. The learning rule is dependent on the length of the SP burst, we considered 2-spike bursts (2 spikes within 15 ms) as well as 4-spike bursts (4 spikes within 45 ms) separately. Note that a given spike can only be part of one burst, and the 4-spike burst takes priority. As such, spike trains were analyzed for bursts every time the SP cell produces a new spike. The new spike and the three preceding spikes are analyzed and (a) if none of them are part of bursts already and (b) the first and last spike are within 45 ms apart of each other, then the group of spikes is considered a 4-spike burst. If they do not constitute a 4-spike burst, then the fourth most recent spike and the fifth most recent spike are analyzed and (a) if neither spike is part of a burst already and (b) the spikes are within 15 ms of each other, then they are considered a 2-spike burst. In this way, no spike that may become part of a 4-spike burst is mistakenly placed in a 2-spike burst, and every spike that cannot be part of a 4-spike burst is checked to see if it can be placed in a 2-spike burst.

When a burst in SP cell *i* is recorded, all the synaptic weights *w_i,s_* are updated according to:
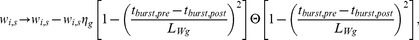
(13)where *g* = 2,4 depends on whether the SP cell burst consists of 2 or 4 spikes, *t_burst,pre_* is the onset time of the pre-synaptic burst, *t_burst,post_* is the onset time of the SP cell burst, *η_g_* is a constant gain term, and *L_Wg_* is the length of the time windows for g-spike SP cell burst. Thus, parameters *η_2_* and *L_W2_* are used for 2-spike bursts while parameters *η_4_* and *L_W4_* are used for 4-spike bursts. The pre-synaptic onset burst times, *t_burst,pre_*, were taken to be the times at which the sinusoidal input to each granule cell reaches a local maximum. A non-associative potentiation rule is also included in order to ensure that not all synaptic weights reach zero due to the aforementioned depression rule. Thus, all the synaptic weights evolve according to:
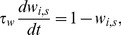
(14)where τ_w_≫1.

Previous studies have shown that neighboring pyramidal cells within the centrolateral and lateral segments tend to display significant correlations between their baseline (i.e. in the absence of stimulation) activities [26], which correlates well with anatomical findings showing significant shared input from peripheral receptor afferents [23]. We mimicked this shared input by decomposing the sources of noise onto both the SP cells as well as the granule cells. Specifically, we have:
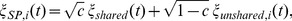
(15)where the noise *ξ_shared_(t)* and *ξ_unshared,i_(t)* have the same statistics as *ξ_SP,i_(t)* except that the former is common between both SP cells while the *ξ_unshared,i_(t)* are independent and identically distributed. Thus, the correlation coefficient between the noise sources to both SP cells is *c*. Similarly, the noise to each granule cell, *ξ_PF,i_(t)*, consists of a component that is inherited from the deep pyramidal cells from which it receives input, *ξ_DP_(t)*, and a component that is independent to each granule cell *ξ_i_(t)*:
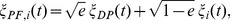
(16)where *ξ_i_(t)* are independent and identically distributed and *e* is the fraction of shared noise with the pyramidal cell. Since anatomical studies have shown that there is almost complete overlap between the feedforward inputs to DPs and SPs within the same column [23], we took *ξ_DP_(t)* = *ξ_SP,i_(t)*. Thus, using eq. (15), we have:
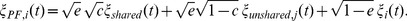
(17)Where *ξ_shared_(t)* is common to all granule cells, *ξ_unshared,j_(t)* is common to all granule cells projecting to pyramidal cell j, and *ξ_i_(t)* is independent across granule cells. Thus, granule cells will tend to display more correlations in their trial-to-trial variabilities when both *c* and *e* are close to 1.

We assumed a homogeneous network for SPs and granule cells and, unless otherwise specified, parameter values used were: *V_threshold_* = −65 mV, *V_rest_* = −68.8 mV, *r_s_* = 0.7 ms, *I* = 0.313µA/cm^2^, *σ* = 0.412 µA/cm^2^, *ρ* = 0.412 µA/cm^2^, κ = 0.21 µA/cm^2^, τ_w_ = 4900 s, *E_leak_* = −68.8 mV, *E_AMPA_* = 0 mV, *E_GABA_* = −68.8 mV, *g_leak_* = 0.14 mS/cm^2^, *g_GABA_* = 0.14 mS/cm^2^, *g_max_* = 0.024 mS/cm^2^, C = 1 µF/cm^2^, *τ_AMPA_* = 5.26 ms, *A* = 0.6, *B* = 2, *D* = 0.7 ms, *E* = 24.5 ms, *γ* = 0.2, *α* = 10.9 µA/cm^2^, *τ* = 7 ms, *L_W2_* = 10 ms, *L_W4_* = 100 ms, *η_2_* = 0.0018, *η_4_* = 0.0036, *c* = 0.25, *e* = 1.

The model was simulated numerically using an Euler-Maruyama algorithm with dt = 0.05 ms. As previous studies have shown that local stimuli did not elicit significant feedback input onto pyramidal cells [22], [24]–[26], [30], we mimicked this stimulation in our model by setting both *g_max_* and *g_GABA_* to zero. For global stimulation, we initially set all synaptic weights to 1 and allowed them to settle to equilibrium over a simulation time of 1000 s (i.e. “training”). These weights were then kept fixed at these values for simulations that were run over 100 trials of 100 sec each that are presented in the results except for Fig. 3. We note that similar results were obtained when the weights were allowed to evolve. For the results presented in Figs. 3A,B, the weights were allowed to vary according to the plasticity rule (eq. 13). For Figs. 3C, 3D, the weights were kept fixed at their values for the corresponding time during training in order to avoid non-stationarities and thus be able to compute the shown quantities.
